# Mass Spectrometric Investigation of Organo-Functionalized Magnetic Nanoparticles Binding Properties toward Chalcones

**DOI:** 10.3390/ma14164705

**Published:** 2021-08-20

**Authors:** Mateusz Pawlaczyk, Rafał Frański, Michał Cegłowski, Grzegorz Schroeder

**Affiliations:** Faculty of Chemistry, Adam Mickiewicz University, 61-614 Poznań, Poland; franski@amu.edu.pl (R.F.); michal.ceglowski@amu.edu.pl (M.C.); schroede@amu.edu.pl (G.S.)

**Keywords:** Fe_3_O_4_ nanoparticles, Schiff base, hybrid materials, adsorption, chalcones, ESI-MS, FAPA-MS

## Abstract

Chalcones are naturally occurring compounds exhibiting multiple biological functions related to their structure. The investigation of complexes formed by chalcones, namely 2′,4′-dihydroxy-2-methoxychalcone (DH-2-MC) and 2′,4′-dihydroxy-3-methoxychalcone (DH-3-MC), with organo-functionalized Fe_3_O_4_ magnetic nanoparticles using mass spectrometric techniques is reported. The magnetic nanoparticles were obtained by the silanization of Fe_3_O_4_ particles with 3-aminopropyltrimethosysilane, which were subsequently reacted with 3-hydroxybenzaldehyde (3-HBA) or 2-pyridinecarboxaldehyde (2-PCA), resulting in the formation of Schiff base derivatives. The formation of their complexes with chalcones was studied using electrospray (ESI) and flowing atmosphere-pressure afterglow (FAPA) mass spectrometric (MS) ionization techniques. The functional nanoparticles which were synthesized using 3-hydroxybenzaldehyde displayed higher affinity towards examined chalcones than their counterparts obtained using 2-pyridinecarboxaldehyde, which has been proved by both ESI and FAPA techniques. For the examined chalcones, two calibration curves were obtained using the ESI-MS method, which allowed for the quantitative analysis of the performed adsorption processes. The presence of Cu(II) ions in the system significantly hindered the formation of material–chalcone complexes, which was proved by the ESI and FAPA techniques. These results indicate that both mass spectrometric techniques used in our study possess a large potential for the investigation of the binding properties of various functional nanoparticles.

## 1. Introduction

The term “chalcone” was implemented into the chemical nomenclature at the end of the 19th century and referred to a structure of benzylidene acetophenone. Along with the discovery and synthesis of chalcone-based structures containing phenyl rings substituted with various functional groups, the term “chalcone” has been broadened and now collectively refers to an entire group of pure chalcone and its multiple derivatives. Such a vocabulary prerogative might be justified by the wide range of chalcones’ biological activities, including anticancer, anti-bacterial, anti-viral, or antioxidative therapeutic effects [1,2]. Although naturally derived or synthetic chalcones are mostly studied for their clinical applicability in the treatment of several diseases, they exhibit interesting structural features, which allow their application in chemical analysis.

In recent years, several chalcones were proved to play the role of non-toxic and eco-friendly corrosion inhibitors due to their ability to substitute water molecules adsorbed on metal surfaces, thus hindering the aggressiveness of aqueous solutions towards an exposed surface [3,4]. The investigated values of energy band gaps of chalcones are also promising for their application in optoelectronics [5]. Among all the analytical applications of chalcones, detection and quantification of several ionic species, metal cations especially drew the largest attention. Such a scientific direction has its source in the intrinsic fluorescent character of chalcones due to their conjugated system of π-electrons of phenyl rings, C=C bond, and C=O bond. Along with the functionalization of the phenyl rings with specific domains, which affords the final planarity of the molecule, chalcone derivatives feature the intramolecular charge transfer (ICT) phenomenon, which makes them a group of ions-sensitive sensors [6]. Incorporating various organic residues to chalcone structures may affect an intensified ICT, and thus increased fluorescence, and may be used as a signaling unit in spectral assays, leading to novel sensors working in “on/off mode” under the presence and absence of the specific ions. For instance, chalcones modified with the naphthalenoxy group via formyl linker demonstrated high selectivity towards Al(III) ions, caused by the presence of three oxygen atoms taking part in the ions coordination [7], while chalcones modified with anthracene and pyridine domains was proved for the selective binding of Pb(II) ions [8]. Chalcones containing pyrene and pyridine pending groups have been tested for specific Ni(II) binding, which triggered its use as a tool for the investigation of Ni-contamination of biological samples using fluorescent microscopic assays [9]. Coumarin-functionalized chalcone was considered as a very efficient chemosensor towards Cd(II) ions, detecting their contamination at nanomolar concentration via colorimetric, fluorometric, and even “naked-eye” tests [10], and 2-hydroxyquinoline-modified chalcone exhibited selectivity towards Fe(III) ions [11]. Such systems have also been studied for highly satisfactory sensing of anions such as fluoride, peroxynitrite, and cyanide. These various detection mechanisms, including enhancing ICT and the formation of adducts, result in changes in the spectral responses [12,13,14]. Moreover, chalcones containing pending groups of boronic acid and dimethylamine were studied as a probe for carbohydrates sensing [15]. Under the addition of sugars, a charge transfer between B(OH)_2_ and NMe_2_ groups intensified, leading to an increase in fluorescence intensity dependent on the concentration of the added carbohydrate.

The affinity of chalcone-based structures towards metal cations might result in the design and creation of new metal-binding materials. Such an approach was proposed by El-Nahass et al., who obtained mesoporous silica KIT-6 functionalized with chalcone isothiocyanate as a sensing material for chosen metals [16]. The sensing potential was proven even at Cd(II), Co(II), or Sb(III) 5 ppb concentration, investigated either by fluorometric assays or the visual effect of the material’s color change from pale yellow to cyan. The binding of the metal cations, especially those of d-block, through their coordination by chalcone residue immobilized on a support surface might also be a convenient approach for the storage and delivery of metal–chalcone complexes. These kinds of complexes are of great interest since cations of transition metals stabilize the coordinating bio-compound and may impact the molecule’s bioactivity, including enhanced antioxidative, anticancer, anti-fungal, or antibacterial effects [17,18,19,20,21]. For instance, polyhydroxy-substituted chalcones complexed with Cu(II) or Zn(II) ions, and amino-derivative of pyridine–chalcone complexed with copper nanoparticles, displayed significant antioxidative potential when tested using the 1,1-diphenyl-2-picrylhydrazyl (DPPH) free radical test [22,23,24]. Significantly increased antimicrobial and anti-fungal effects were also proved for various chalcone–transition metal complexes in reference to free chalcone ligands [25,26,27].

The high affinity of chalcones towards cations complexation, and the elevated therapeutic effects of such coordination complexes, have directed our attention towards the implementation of functional materials able to bind transition metal ions and/or chalcones, which may be applied as platforms for simultaneous chalcones’ scavenging and transport of the obtained chalcone-metal bioactive complexes. Therefore, this study focuses on preparing magnetically susceptible supports functionalized with different Schiff bases as metal chelators, with their further multiway application as storage and delivery tools of chalcones or metal–chalcone complexes. The proposed method involves investigating sorption processes with MS analyses, by using two different ionization sources: electrospray (ESI) and flowing atmosphere-pressure afterglow (FAPA) modes.

## 2. Materials and Methods

### 2.1. Chemicals

All of the reagents used, namely 3-hydroxybenzaldehyde (3-HBA; ≥99%), 2-pyridinecarboxaldehyde (2-PCA; 99%), 2′,4′-dihydroxy-2-methoxychalcone (DH-2-MC; 97%), 2′,4′-dihydroxy-3-methoxychalcone (DH-3-MC; 97%), and copper(II) perchlorate hexahydrate (98%), were obtained from Sigma Aldrich (St. Louis, MO, USA). All of the solvents were of purity grade p.a. and were used without any purification. Methanol (MeOH) and hydrochloric acid (HCl) were purchased from Stanlab (Lublin, Poland), dichloromethane (DCM) from Eurochem BGD (Tarnów, Poland), and ethyl alcohol absolute (EtOH) from POCH (Gliwice, Poland).

### 2.2. Instruments

The obtained Schiff base-functionalized materials were characterized with FT-IR analysis using the Bruker IFS 66v/S instrument (Bremen, Germany). The spectra were recorded between 4000 and 400 cm^−1^, using KBr pellets as analytic medium. The complexes of hybrid materials with Cu(II) ions were investigated using X-ray fluorescence (XRF) measurements performed using Malvern PANalytical B.V. (Almelo, Netherlands) apparatus. The spectrometer was equipped with an X-ray source-rhodium vacuum tube. The analyses lasted 200 s, and were conducted with the X-ray tube voltage of 13 kV, automatically adopted current, and Al-filter excluding lighter elements. The planned multiple UV-Vis measurements were conducted using Agilent 8453 spectrophotometer (Santa Clara, USA). The solutions were analyzed using standard poly (methyl methacrylate) (PMMA) cuvettes of optical length of 10 mm, in the wavelength range of 250–1000 nm (resolution: 1 nm). The measurements were performed in triplicate. 

All of the MS spectra were obtained using an amaZon SL ion trap (Bruker, Bremen, Germany) mass spectrometer in the mass range between 100 and 1000 *m*/*z*, which operated under high vacuum, utilizing helium and nitrogen as the cone and the desolvating gases, respectively. The solutes remaining after the adsorption processes were analyzed in the electrospray ionization source mode (ESI-MS) operating in infusion mode. For these measurements, the source and the desolvation temperatures were set at 80 and 250 °C, respectively, whereas the capillary voltage and end plate offset were set at −4.5 and −0.5 kV, respectively. The solutions were injected into the ionization source at a flow rate of 10 μLmin^−1^ using a syringe pump. The complexes of materials with adsorbed chalcones were further studied using V-shaped flowing atmosphere pressure afterglow (V-FAPA) ambient plasma ion source (ERTEC, Wroclaw, Poland). The generated helium plasma stream was located axially in a distance of approximately 10 cm from the inlet of the spectrometer. The samples directly introduced to the plasma stream or 5 mm below the stream were also heated on a crucible, with a temperature control up to 250 °C, and a heating rate of 3 °C s^−1^. The non-covalently attached analytes, which underwent thermally induced ionization in the plasma stream, were directly infused to the spectrometer’s analyzer.

### 2.3. Synthesis of Schiff Base-Modified Fe_3_O_4_ Nanoparticles

The support used were nanoparticles of magnetite encapsulated within silica matrix and further functionalized with 3-aminopropyltrimethoxysilane (APTMS), which was performed using the procedure described earlier [28]. Obtained particles of Fe_3_O_4_-SiO_2_-NH_2_ were further reacted with two aromatic aldehydes to obtain magnetite particles modified with surface Schiff base domains. Therefore, 1.38 g of the material was dispersed in 50 mL of ethanol and heated in an ultrasound bath to 65 °C maintaining a continuous sonification. Then, a solution of 3-hydroxybenzaldehyde (0.183 g; 1.5 mmol) or 2-pyridinecarboxaldehyde (0.161 g; 1.5 mmol) and 60 μL of concentrated HCl in 40 mL of ethanol was added dropwise. The sonification at elevated temperature was performed for 4 h, and shaken at r.t. for 16 h. Afterward, the materials were separated using magnetic decantation, washed with EtOH and DCM (2 × 15 mL), and dried under vacuum at 50 °C for 8 h. The resulting dark solids denoted as Fe_3_O_4_-3-HBA and Fe_3_O_4_-2-PCA, respectively, were ground. For further studies, a part of the obtained materials was complexed with Cu(II) ions. Briefly, 400 mg of Fe_3_O_4_-3-HBA or Fe_3_O_4_-2-PCA were mixed with 100 mL of 4 mM solution of Cu(ClO_4_)_2_ in distilled water at r.t for 24 h. The obtained [Fe_3_O_4_-3-HBA]Cu(II) and [Fe_3_O_4_-2-PCA]Cu(II) complexes were magnetically separated, washed three times with distilled water (50 mL), and dried under vacuum in a desiccator at r.t. overnight.

### 2.4. Synthesis of 2′,4′-Dihydroxy-2-Methoxychalcone (DH-2-MC) Complex with Cu(II) Ions

To a refluxing solution of Cu(ClO_4_)_2_ (185.3 mg; 0.5 mmol) in 20 mL of ethanol, a solution of DH-2-MC (135.2 mg; 0.5 mmol) in 20 mL of ethanol was added dropwise. The mixture was further refluxed for 4 h, with subsequent cooling to r.t., evaporation of the solvent, and precipitation with distilled water. The resulting yellow-red solid of [DH-2-MC]Cu(II) was centrifuged, washed with distilled water, and dried under vacuum at r.t. for 8 h. ESI-MS(+): *m*/*z* 271.17 [DH-2-MC + H]^+^; 293.17 [DH-2-MC + Na]^+^; 332.07 [[DH-2-MC]Cu(II)-H]^+^; 350.09 [[DH-2-MC]Cu(II)-H + H_2_O]^+^; 373.12 [[DH-2-MC]Cu(II)-2H + Na]^+^; 602.24 [[DH-2-MC]_2_Cu(II)-H]^+^.

### 2.5. Adsorption Experiments 

#### 2.5.1. Adsorption of Pure Chalcones on the Fe_3_O_4_-Schiff Base Materials

A series of approximately 15 mg samples of Fe_3_O_4_-3-HBA or Fe_3_O_4_-2-PCA were added to a series of DH-2-MC or DH-3-MC solutions of concentrations 10^−8^, 10^−7^, 10^−6^, 10^−5^, and 10^−4^ M using a mixture MeOH:H_2_O (2:1) as a solvent. The material-chalcone mixtures were shaken for 24 h at r.t. Afterward, the solids were centrifuged, and the solutes were filtered using syringe filters prior to their analysis with the ESI-MS measurements. Concentrations of the chalcones remaining in the solutions after the adsorption processes were established using pre-prepared calibration curves (signal intensity vs. concentration) using DH-2-MC and DH-3-MC MeOH:H_2_O (2:1) solutions at the following concentrations: 10^−8^, 10^−7^, 5 × 10^−6^, 10^−6^, 5 × 10^−5^, 10^−5^, and 10^−4^ M. Moreover, the 50 mg samples of both materials incubated in the most concentrated chalcone solutions according to above-described method were subsequently dried and subjected to direct FAPA-MS analysis.

#### 2.5.2. Adsorption of Pure Chalcones on the [Fe_3_O_4_-Schiff base]Cu(II) Materials 

To MeOH:H_2_O, (2:1) solutions of DH-2-MC or DH-3-MC of 10^−7^, 5 × 10^−6^, and 10^−4^ M, 15 mg samples of [Fe_3_O_4_-3-HBA]Cu(II) and [Fe_3_O_4_-2-PCA]Cu(II) were added. The mixtures were shaken for 24 h at r.t and then separated as described in point 2.5.1. The remaining solutes were further analyzed using the ESI-MS technique, leading to the establishment of the percentages of the chalcones adsorbed. Furthermore, the dried material-Cu(II)-chalcones complexes were subjected to FAPA-MS analysis, in order to record the spectra of chalcones desorbed from the complexes. 

#### 2.5.3. Adsorption of [DH-2-MC]Cu(II) Complex on the Fe_3_O_4_-Schiff Base Materials

Samples of 15 mg of Fe_3_O_4_-3-HBA and Fe_3_O_4_-2-PCA were added to 5 mL of a solution of [DH-2-MC]Cu(II) (obtained as: 5 mg of complex + 1 mL EtOH + 9 mL MeOH:H_2_O (2:1)). The mixtures were shaken at r.t. overnight, and the resulting solids were magnetically decanted, dried, and subjected to FAPA-MS analysis.

## 3. Results and Discussion

### 3.1. Synthesis and Characterization of Schiff Base-Modified Hybrid Materials

**Figure 1:**
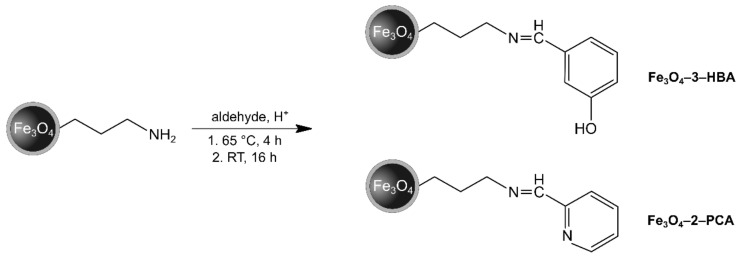
Synthesis of the Schiff base-functionalized materials.

**Figure 2:**
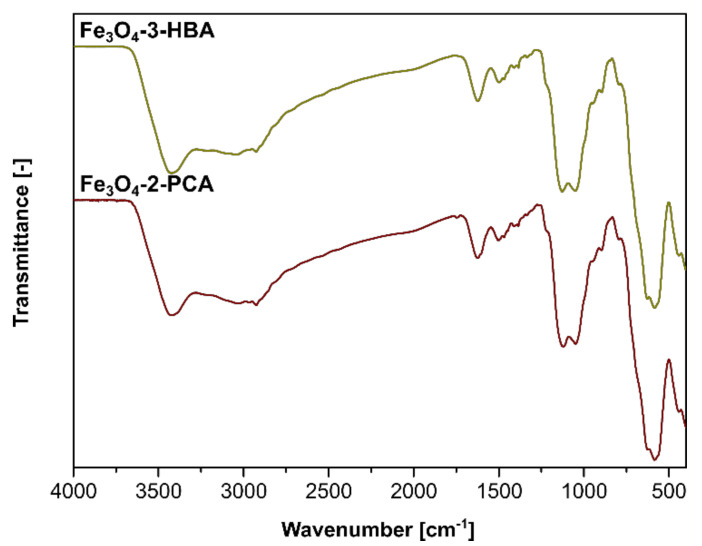
The FT-IR spectra of the synthesized materials.

The designed materials consisting of magnetite nanoparticles as a core and two different Schiff bases as functionalizing agents were obtained using a classic condensation protocol between the free pendant -NH_2_ group attached to the support and -CHO group of the chosen aldehydes (3-hydroxybenzaldehyde or 2-pyridinecarboxaldehyde) in an acidic environment, as illustrated in Figure 1. The obtained materials Fe_3_O_4_-3-HBA and Fe_3_O_4_-2-PCA were characterized using FT-IR spectroscopy, of which spectra are presented in Figure 2. For both materials, the spectra present the most informative signal at 1624 cm^−1^, which is related to the stretching of -C=N-, confirming the successful formation of Schiff base on the materials’ surface. Moreover, a sequence of signals between 1500 and 1400 cm^−1^ in both spectra corresponds to the stretching of C=C_(arom)_ domains of the aryl aldehydes used. The applied support of Fe_3_O_4_ nanoparticles encapsulated within a thin silica layer, further functionalized with silane amino-derivative, triggered the appearance of several signals in the materials’ spectra at 3423, 1050, and 585 cm^−1^, which are connected to the stretching of Fe-O, the stretching of Si-O-Si, and the stretching of N-H, respectively.

### 3.2. Adsorption of the Chalcones on the Schiff Base-Modified Materials

**Figure 3:**
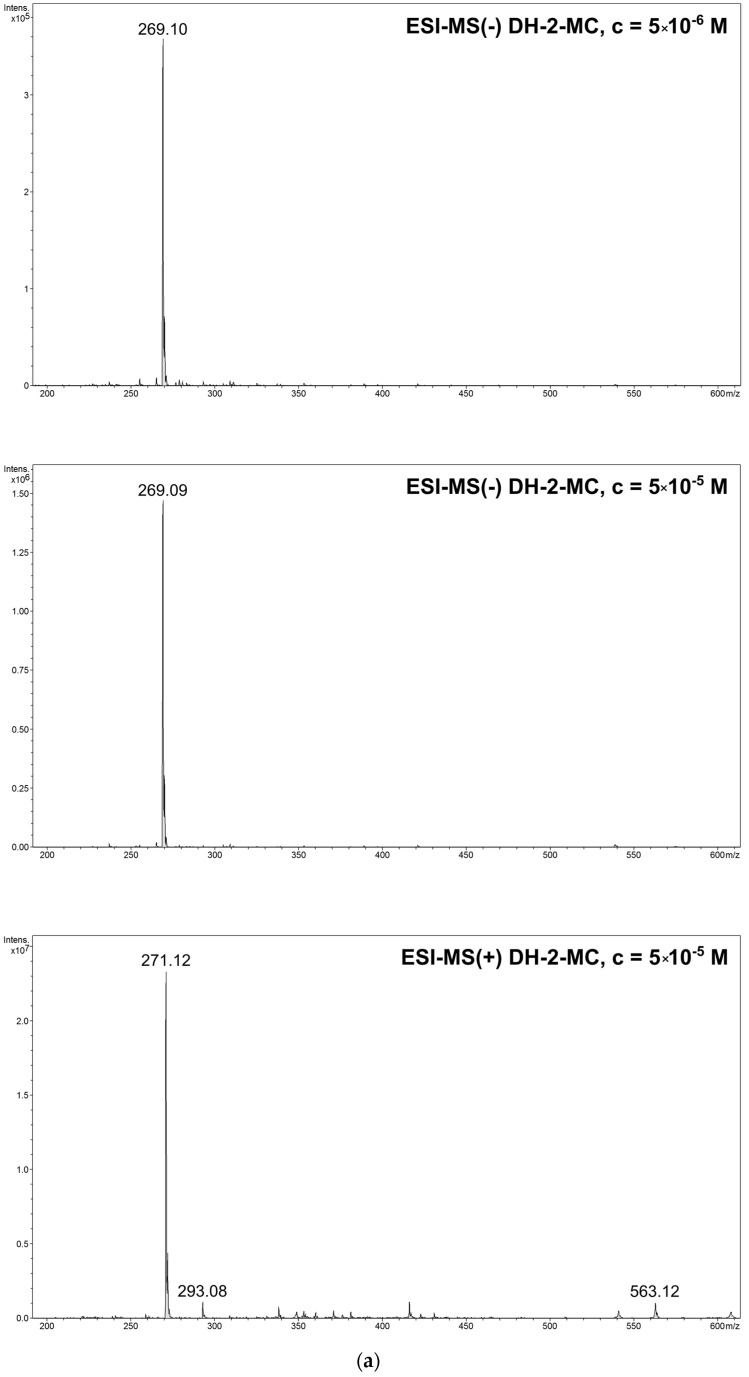

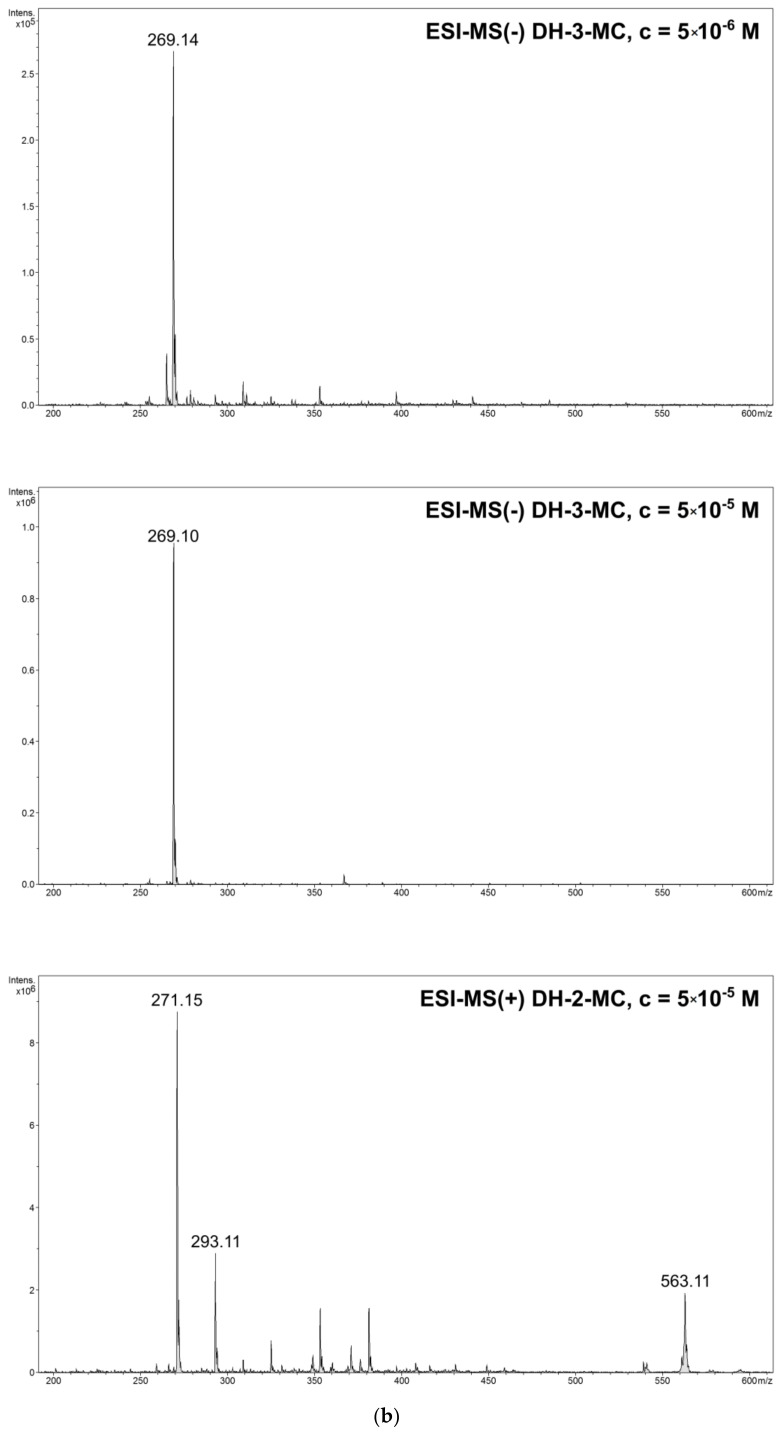
The ESI-MS spectra of (**a**) DH-2-MC and (**b**) DH-3-MC, performed in negative (**top** and **middle**) and positive mode (**bottom**).

The adsorption of pure hydroxymethoxy derivatives of chalcones (DH-2-MC and DH-3-MC) was monitored using electrospray ionization mass spectrometry (ESI-MS) measurements in negative ions mode by the observation of single analytes’ molecular peaks at *m*/*z* 269. Although chalcones are prone to cationization, their spectra in positive ions mode not only displayed a molecular peak at *m*/*z* 271, but also sodium-adduct and dimeric signals at *m*/*z* 293 and 563, respectively (Figure 3), which causes serious obstacles in quantitative analysis.

**Figure 4:**
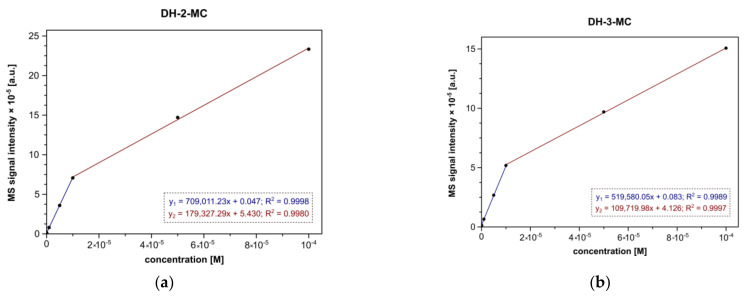
The ESI-MS calibration curves obtained for both chalcone analytes: (**a**) DH-2-MC and (**b**) DH-3-MC.

The application of MS methods has previously been described as an efficient technique for the characterization and/or quantification of several hydroxychalcones [29]. Since the studied chalcones are the substituent isomers (difference in the position of methoxy group), investigation of their adsorption on the hybrid materials was conducted in single-component solutions. To perform reliable quantification of the adsorption’s progress, the dependence of the molecular signal intensity of ESI-MS analysis performed in negative mode (*m*/*z* 269) on the concentration of pure chalcones was performed. The prepared calibration curves for both DH-2-MC and DH-3-MC involved an analysis of seven water-ethanol solutions of the concentrations ranging between 10^−8^ and 10^−4^ M, which are presented in Figure 4. For both chalcones, the signal intensity is proportional to the concentration in two intervals: below and above the chalcone concentration of 10^−5^ M. Such a phenomenon is related to the appearance of chalcone-dimer signals in the spectra in higher concentrations, leading to the less proportional increase in the analytical signal *m*/*z* 269. Therefore, both calibration curves were described with two linear plots.

**Figure 5:**
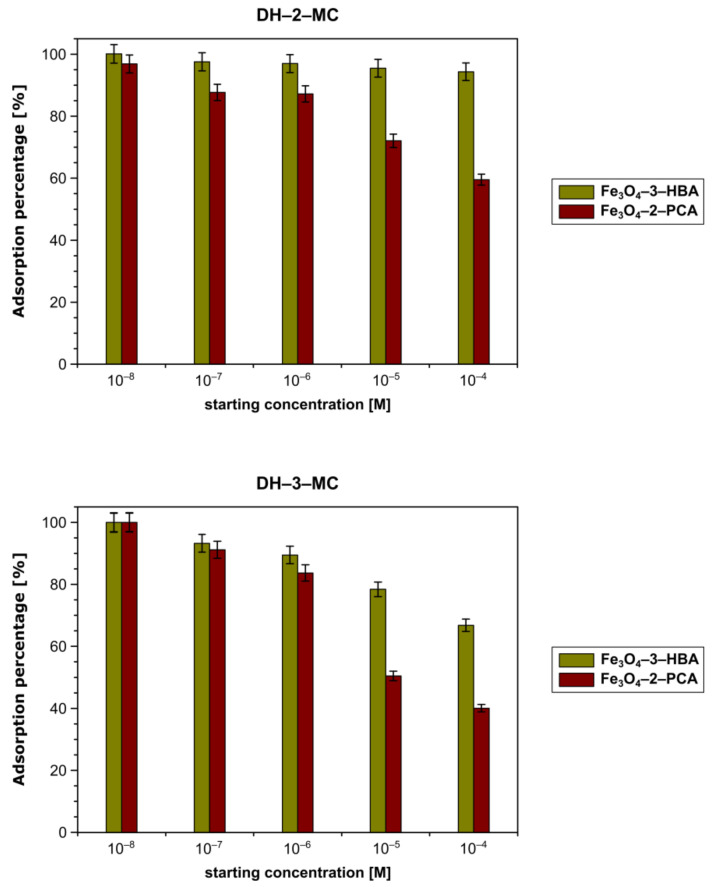
The percentages of (**top**) DH-2-MC and (**bottom**) DH-3-MC adsorption by two Schiff base-functionalized magnetic hybrid materials.

**Figure 6:**
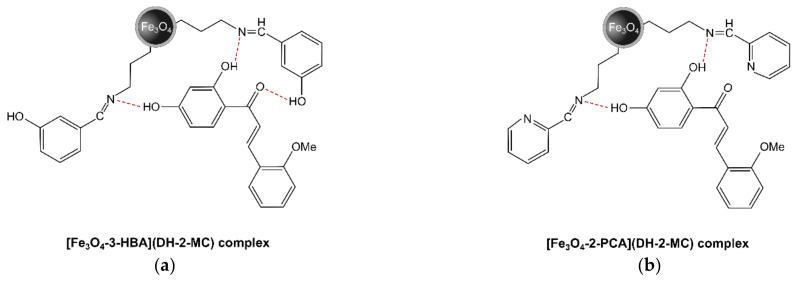
The proposed structures of complexes formed by hydrogen bonds (red dotted lines) between DH-2-MC and the hybrid materials containing Schiff base residues: (**a**) 3-HBA and (**b**) 2-PCA.

The progress of the chalcones adsorption from water–ethanol solutions by the hybrid materials Fe_3_O_4_-3-HBA and Fe_3_O_4_-2-PCA was investigated based on *m*/*z* 269 signal intensity in the ESI-MS spectra of solutes remaining after the adsorption processes. The percentages of chalcones removal calculated on the basis of the pre-performed ESI-MS calibration curves, presented in Figure 5, show that both materials have satisfactory adsorptive properties towards the chosen analytes, emphasizing the binding properties of the material containing hydroxyphenyl residue. The material displayed highly significant removal of DH-2-MC and DH-3-MC, reaching the adsorption percentages of 94.25% and 66.82%, respectively, in the most concentrated analyte’s solution. For the material containing pendant pyridine ring, these values reached 59.45% and 40.07%, respectively, showing lower but still satisfactory adsorption. Such a result might be driven mainly by the presence of the pendant hydroxyl group in material Fe_3_O_4_-3-HBA, which stabilizes chalcones binding through the formation of hydrogen bond with chalcones’ carbonyl group, leading to more effective adsorption (Figure 6). The localization of methoxy substituent in chalcone structure also impacts their ability to diffuse between the pendant Schiff base residues on the material’s surface, which is related to the more significant steric hindrance caused by *meta*-substitution than that for *ortho*-substitution.

**Figure 7:**
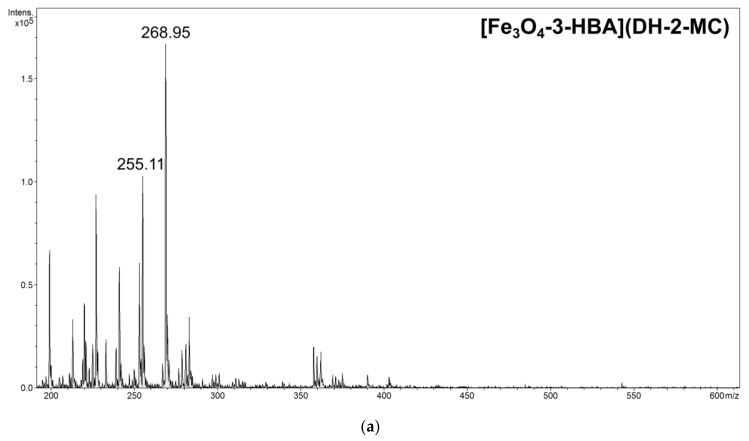

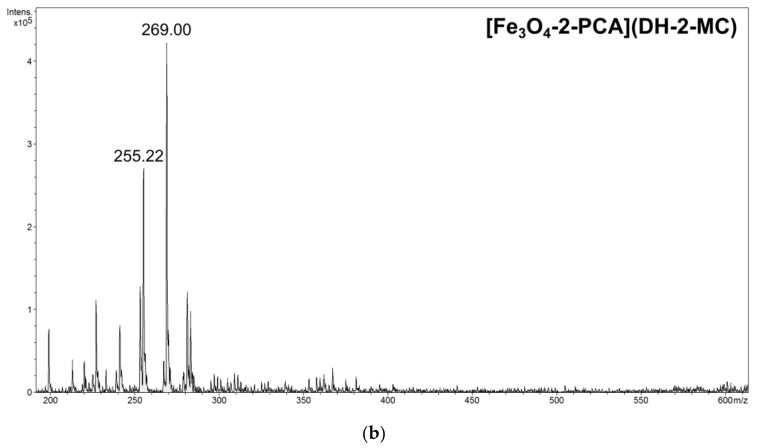
Exemplary FAPA-MS spectra of DH-2-MC thermally desorbed from 50 mg samples of its complexes with (**a**) Fe_3_O_4_-3-HBA and (**b**) Fe_3_O_4_-2-PCA.

**Figure 8:**
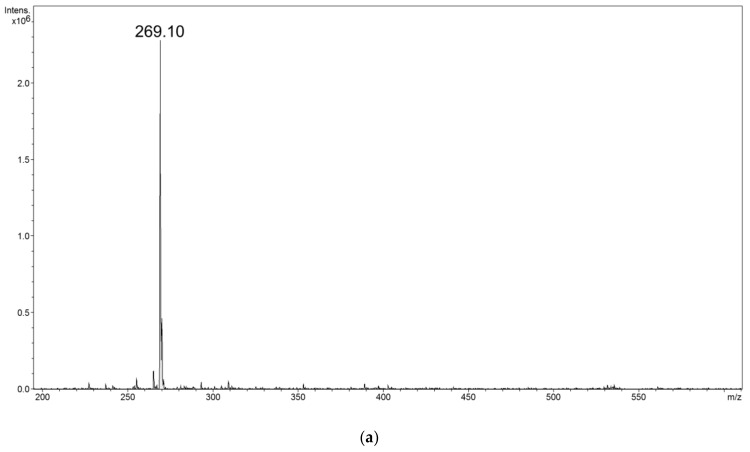

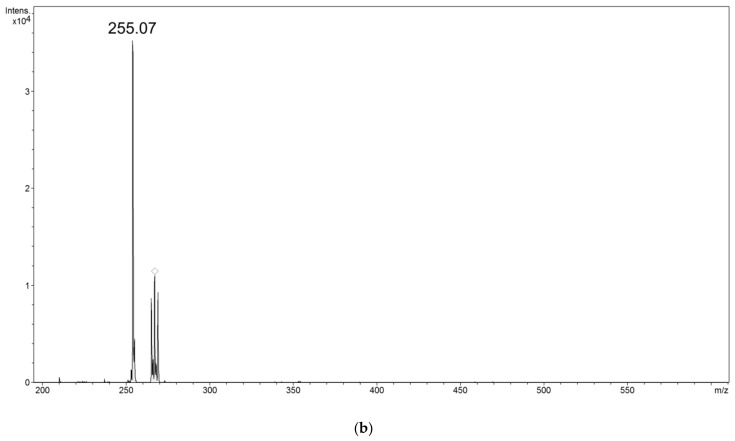
The spectra of DH-2-MC recorded in (**a**) FAPA-MS and (**b**) FAPA-MS^2^ modes. The chalcone’s spectrum after applying the low collision energy to the molecular signal *m*/*z* 269 shows the main fragmentation signal at *m*/*z* 255, which is related to the chalcone’s fragmentation ion after the loss of -CH_3_ group of methoxy domain.

Even though the above-described structural features, either of the chalcones or the functionalizing agents, impact the adsorption processes, the formed hybrid material–chalcone complexes were analyzed using mass spectrometry working in flowing atmosphere-pressure afterglow ionization mode (FAPA-MS). The main objective of the performed FAPA-MS analysis was to demonstrate the presence of the non-covalently adsorbed chalcones on the studied materials, and their ability of being detected using this kind of direct analysis (a qualitative analysis). This ionization source enables a highly sensitive and fast analysis, including the direct analysis of liquids and solids, as well as the indirect analysis of analytes adsorbed on the matrix, which overcome the limits or difficulties ascribed for ESI-MS analysis. Figure 7 presents the spectra of chalcones thermally desorbed from exemplary material–chalcone complexes using FAPA-MS technique, which proves the ionization of non-covalently bound chalcones in the stream of helium plasma. The spectra indicate two main signals corresponding to molecular and fragmentation ions *m*/*z* 269 and 255, respectively. To prove the observation of a fragmentation signal in the described spectra, a tandem mass spectrum (FAPA-MS^2^) of pure DH-2-MC was recorded, which is presented in Figure 8. The main fragmentation signal observed after low energy collision-induced dissociation is the signal at *m*/*z* 255, corresponding to the chalcone’s fragment after the loss of methyl group from methoxy domain. Moreover, both molecular and fragmentation signals in the FAPA-MS spectra of the chalcones released from material–chalcone complexes even reached 4 × 10^5^, which, in comparison to the pre-performed calibration curves, indicate an effective chalcones binding on the materials (the intensities correspond to the chalcones’ concentration of ∼1 × 10^−5^ M). 

**Figure 9:**
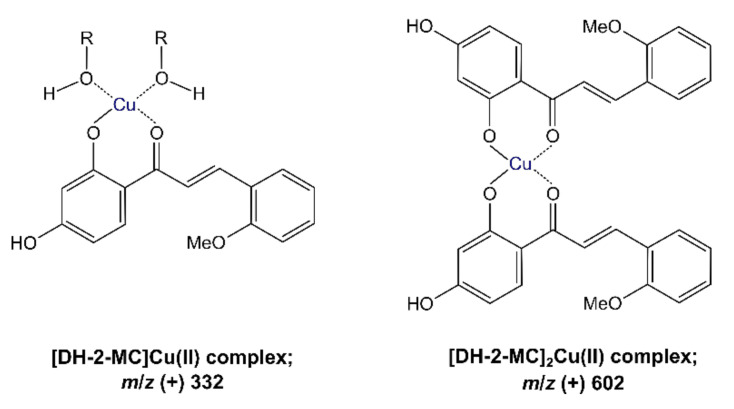
The proposed mono- and dimeric structures of DH-2-MC and Cu(II) complex. In the monomeric complex, R-OH molecules may appear as additional ligands (R = H, Me, or Et, related to the synthesis and analysis conditions), which do not interfere in its MS analysis.

Moreover, both chalcones and Schiff bases are known for their ability to form complexes with metal cations, especially divalent cations of transition metals [30,31]. The complexation of one of the systems, either Schiff base or chalcone, may significantly impact the interactions between Schiff base-functionalized materials and chalcones. Therefore, the following study included an investigation of the Cu(II) ions presence in the system on the hybrid materials’ adsorptive efficiency towards chalcones. The studies involved two different approaches: (1) investigation of interactions between pure chalcones (DH-2-MC and DH-3-MC) and the materials complexed with Cu(II); and (2) investigation of interactions between the hybrid materials and the synthesized DH-2-MC-Cu(II) complex. The proposed structures of the formed and ESI-MS analyzed complex of DH-2-MC and Cu(II) ions are illustrated in Figure 9. On the other hand, the complexation of both Fe_3_O_4_-based materials with copper ions was proved by X-ray fluorescence (XRF) analysis, the spectra of which displayed a signal corresponding to the presence of Cu.

**Figure 10:**
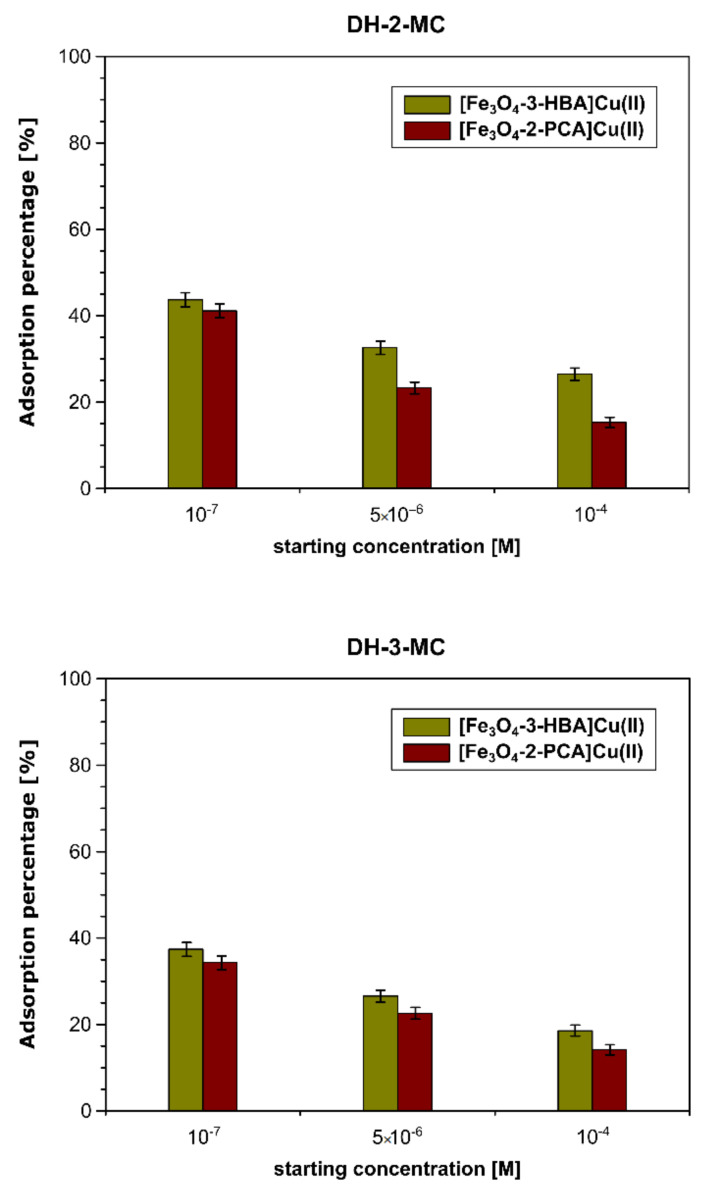
The percentages of the chalcones adsorbed on the hybrid materials complexed with Cu(II) ions for: (**top**) DH-2-MC and (**bottom**) DH-3-MC, investigated using ESI-MS analysis.

Furthermore, [Fe_3_O_4_-3-HBA]Cu(II) and [Fe_3_O_4_-2-PCA]Cu(II) were tested for their ability to adsorb the studied chalcones using ESI-MS measurements of the solutes remaining after the adsorption process. The percentages of the chalcones adsorbed by the materials, calculated on the basis of the previously presented calibration curves, are presented in Figure 10.

**Figure 11:**
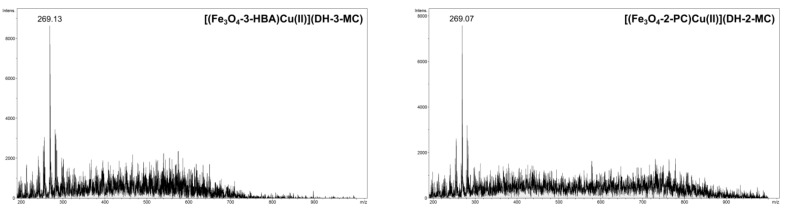
The FAPA-MS spectra of the complexes of chalcones with Cu(II)-complexed Schiff base-functionalized materials.

The Cu(II)-complexed hybrid materials demonstrated significantly lower adsorption efficiency towards chalcones than the bare Schiff base-functionalized materials, reaching adsorption percentages of 42% and 36% for the adsorption of DH-2-MC and DH-3-MC, respectively, from their most diluted solutions (10^−7^ M). Both materials also exhibited a similar trend of an adsorption efficiency decrease as the analytes’ concentration increased, leading to an adsorption of only 14.11%–26.47% of analytes for 10^−3^ M solutions, which is consistent with the performed FAPA-MS measurements of the complexes of hybrid material-Cu(II) with chalcones (Figure 11). The intensities of the signal corresponding to the adsorbed chalcones at *m*/*z* 269 are several times lower than those obtained for non-copper-complexed materials, reaching only ∼7 × 10^3^. Such results indicate the utilization of Schiff bases’ binding domains for the complexation of Cu(II) ions, hindering their attractive interactions with the chalcone structure.

**Figure 12:**
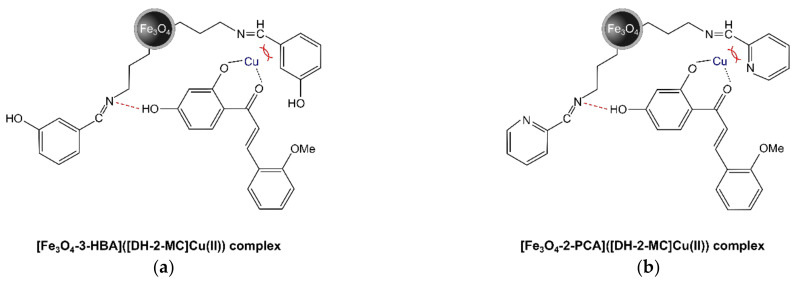
The proposed presentation of decreased interactions between the Cu(II)-complexed hybrid materials containing (**a**) 3-HBA or (**b**) 2-PCA as a pendant residue and DH-2-MC, triggered by a steric hindrance (red brackets).

A similar relationship was observed for the experiments involving the adsorption of the synthesized complex of DH-2-MC and Cu(II) on the hybrid materials. The performed FAPA-MS analyses of the adsorbents after their incubation in DH-2-MC-Cu(II) solution showed no significant signals corresponding to the complex. Such a phenomenon is explainable, taking both the deactivation of hydrogen bonding domains in DH-2-MC and the steric hindrance into account (Figure 12). Therefore, the complexation of chalcones with metal cations leads to their inability to be non-covalently bound by Schiff base residues.

## 4. Conclusions

To conclude, we have shown that mass spectrometry is a universal and reliable tool that allows the investigation of complexes formed by chalcones with functional magnetic nanomaterials. The obtained materials were primarily characterized with FT-IR technique, whereas mass spectrometric analyses were performed using ESI and FAPA-MS. For both of the examined chalcones, two calibration curves were obtained in the concentration range of 10^−8^–10^−4^ M. For both of the chalcones, a single pseudo-molecular ion of *m*/*z* 269 was used as a reference signal corresponding to the formation of deprotonated form of both of the chalcones studied [M–H]^−^. The percentages of the chalcones removal were higher for Fe_3_O_4_-3-HBA, indicating that this material possesses higher adsorption properties than Fe_3_O_4_-2-PCA. This effect is connected with the presence of the hydroxy group in the phenyl ring, which presumably forms stronger hydrogen bonds and interactions than the ones formed by the pyridine ring present in the Fe_3_O_4_-2-PCA structure. Moreover, we have also shown that the *ortho*-substituted chalcones possessed higher adsorption properties towards examined materials than their *meta* counterparts, which is probably connected with differences in a steric hindrance. The presence of Cu(II) cations in the system greatly reduced the amount of complexes formed by chalcones with magnetic materials, proving that copper exhibits a very high affinity to both of these materials. Finally, we have shown that FAPA-MS is a versatile tool which enables tracking for the formation of organic complexes formed by solid adsorbents. The system developed in our research group offers easy operation, rapid analysis, and no sample preparation, which is of high importance for material chemistry.

## Data Availability

The data presented in this study are available on request from the corresponding author.
